# Endoplasmic Reticulum Stress-Associated Neuronal Death and Innate Immune Response in Neurological Diseases

**DOI:** 10.3389/fimmu.2021.794580

**Published:** 2022-01-10

**Authors:** Mingming Shi, Yan Chai, Jianning Zhang, Xin Chen

**Affiliations:** ^1^ Department of Neurosurgery, Tianjin Medical University General Hospital, Tianjin, China; ^2^ Tianjin Neurological Institute, Key Laboratory of Post-trauma Neuro-Repair and Regeneration in Central Nervous System, Ministry of Education, Tianjin, China; ^3^ Department of Neurosurgery, Tianjin Key Laboratory of Injuries, Variations and Regeneration of Nervous System, Tianjin, China

**Keywords:** neuronal death, inflammatory response, neurological diseases, endoplasmic reticulum stress, proteostasis, unfolded protein response

## Abstract

Neuronal death and inflammatory response are two common pathological hallmarks of acute central nervous system injury and chronic degenerative disorders, both of which are closely related to cognitive and motor dysfunction associated with various neurological diseases. Neurological diseases are highly heterogeneous; however, they share a common pathogenesis, that is, the aberrant accumulation of misfolded/unfolded proteins within the endoplasmic reticulum (ER). Fortunately, the cell has intrinsic quality control mechanisms to maintain the proteostasis network, such as chaperone-mediated folding and ER-associated degradation. However, when these control mechanisms fail, misfolded/unfolded proteins accumulate in the ER lumen and contribute to ER stress. ER stress has been implicated in nearly all neurological diseases. ER stress initiates the unfolded protein response to restore proteostasis, and if the damage is irreversible, it elicits intracellular cascades of death and inflammation. With the growing appreciation of a functional association between ER stress and neurological diseases and with the improved understanding of the multiple underlying molecular mechanisms, pharmacological and genetic targeting of ER stress are beginning to emerge as therapeutic approaches for neurological diseases.

## Introduction

The endoplasmic reticulum (ER) is the largest tubular-reticular organelle. It plays essential roles in multiple cellular processes, including calcium homeostasis, lipid synthesis, and the synthesis, folding, maturation, and trafficking of more than one-third of the cellular proteome (1, 2). Multiple disturbances can cause ER proteins to fail to fold into the correct form. Fortunately, the cell has intrinsic quality control mechanisms that discard misfolded/unfolded proteins, such as chaperone-mediated folding (3) and ER-associated degradation (ERAD) (4). However, when these control mechanisms fail, misfolded/unfolded proteins accumulate in the ER lumen. One consequence of the abnormal accumulation of misfolded/unfolded proteins inside the ER lumen is the generation of ER stress, which subsequently triggers a rapid and coordinated biochemical response that is linked to the maintenance of cellular proteostasis; this adaptive process is known as the unfolded protein response (UPR) (5, 6). The UPR is a sophisticated mechanism that can be divided into three types of effector functions: adaption, alarm, and death (7). The initial intent of the UPR when activated by ER stress is restoration of homeostasis and normal ER function. However, when this adaptive mechanism is overwhelmed, such as when ER stress is intense or prolonged, intracellular cascades of death and inflammation are induced (8, 9). Aberrant aggregation of misfolded proteins and concomitant induction of ER stress have been reported to be associated with neuronal death and the inflammatory response in many neurological diseases and implicated in disorders ranging from acute central nervous system (CNS) injuries to neurodegenerative diseases (10, 11). Emerging evidence demonstrates that ER stress plays an essential role in the pathophysiology of neurological diseases; however, the specific mechanisms by which ER stress determines neuronal fate and inflammatory response are not yet understood. In this review, we discuss recent advances in the understanding of the functional links between ER stress and neuronal death and the innate immune responses in the CNS, highlighting their critical roles in the pathogenesis of various neurological diseases and emerging therapeutic opportunities for drug discovery.

## Unfolded Protein Response Signal Transduction Mechanisms

**Figure 1:**
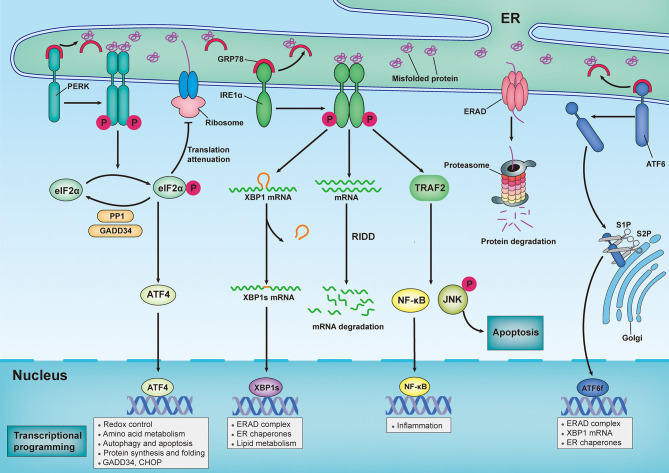
Sensing and responding to endoplasmic reticulum stress: canonical roles of unfolded protein response. In response to an increasingly accumulation of misfolded proteins in endoplasmic reticulum (ER) lumen, three sensors that located in ER membrane — inositol-requiring enzyme 1α (IRE1α), protein kinase RNA-like ER kinase (PERK) and activating transcription factor 6 (ATF6) — provoke unfolded protein response (UPR). Under homeostatic conditions, ER-resident protein chaperone glucose regulated protein 78 (GRP78) interacts with these ER stress sensors to restrain their activation. However, the excessive accumulation of misfolded proteins recruit GRP78 away from all three ER stress sensors, leading to activation of downstream signal transduction pathways. Upon ER stress, PERK undergoes its dimerization and autophosphorylation to phosphorylate eukaryotic translation initiator 2α (eIF2α), which then selectively increases translation of activating transcription factor 4 (ATF4). ATF4 modulates the expression of genes involved in redox control, amino acid metabolism, autophagy, apoptosis, and protein synthesis and folding. Additionally, Phosphorylated eIF2α (p-eIF2α) prevents ribosome assembly, which results in a translational block. Once ER stress is resolved, p-eIF2α is dephosphorylated by the GADD34-protein phosphatase 1 (PP10) complex to restore protein translation. In response to ER stress, IRE1α oligomerizes and promote autophosphorylation, eliciting RNase activity to splice the mRNA of x-box-binding protein 1 (XBP1). Spliced XBP1(XBP1s) mRNA codes for the functionally active proteins of XBP1s, which translocated into nuclear and subsequently induces the transcription of various genes that are involved in protein folding, lipid metabolism and ER-assisted protein degradation (ERAD). In addition, the RNase activity of IRE1α can also degrades a subset of mRNAs in a process termed regulated IRE1α-dependent decay (RIDD). By interacting with adaptor protein TNF receptor-associated factor 2 (TRAF2), IRE1α can also activate c-Jun N-terminal kinase (JNK) and nuclear factor κB (NF-κB) pathways, thereby modulating inflammation and apoptosis. Upon ER stress, ATF6 is transported to Golgi, where it is cleaved by Site-1 protease (S1P) and Site-2 protease (S2P), releasing its active cytosolic fragment (ATF6f) that functions as a transcription factor. ATF6f induces genes required for ERAD and modulates the XBP1 mRNA levels and ER chaperone expression.

In metazoans, three trans-ER membrane proteins sense the aberrant accumulation of misfolded/unfolded proteins and provoke a series of signal transduction pathways to modulate transcriptional and translational programs that combat ER stress (**Figure 1**). The three trans-ER membrane stress sensors that exhibit this coordinated action are inositol-requiring enzyme 1α (IRE1α), protein kinase RNA-like ER kinase (PERK), and activating transcription factor 6 (ATF6) (12). Under homeostatic conditions, the luminal domains of these ER stress sensors are maintained in an inactive state through their interactions with the ER-resident protein chaperone glucose-regulated protein 78 (GRP78; also known as BiP) (13). However, GRP78 has a higher affinity for misfolded proteins than for ER stress sensors; therefore, the excessive accumulation of misfolded/unfolded proteins leads to recruitment of GRP78 away from all three ER stress sensors, permitting the activation of downstream signaling pathways (13). Additionally, misfolded/unfolded proteins can directly bind to PERK and IRE1α, leading to activation of the UPR *via* a ligand–receptor-type interaction (14–16).

IRE1α has been widely reported to be involved in cell death and inflammation in various neurological diseases (6). IRE1α is the most evolutionarily conserved ER stress transducer and contains both kinase and endoribonuclease (RNase) domains. Upon release from GRP78, IRE1α undergoes dimerization in the ER membrane and autophosphorylation, which elicits RNase activity for splicing the mRNA of X-box-binding protein 1 (XBP1) (17). Spliced XBP1 (XBP1s) enters the nucleus and subsequently induces the transcription of various genes involved in protein folding, quality control mechanisms, and ERAD. Additionally, the RNase activity of IRE1α can degrade a subset of mRNAs and microRNAs through a process known as regulated IRE1α-dependent decay (RIDD), which modulates various biological processes including stress mitigation, inflammation, and apoptosis (18, 19). Activation of IRE1α kinase results in recruitment of the adaptor protein TNF receptor-associated factor 2 (TRAF2), which mediates signaling crosstalk with other stress pathways, including the mitogen-activated protein (MAP) kinase (MAPK) pathway and the nuclear factor kappa-light-chain-enhancer of activated B cells (NF-κB) pathway (20). Collectively, IRE1α plays a dual role in the response to ER stress, primarily through XBP1s-mediated adaptative proteostasis, the MAP kinase pathway, RIDD, and NF-κB pathway-mediated apoptosis and inflammation.

Additionally, the PERK-mediated arm of the UPR has been reported to play an important role in various neurological diseases (21). Similar to IRE1α, PERK undergoes dimerization and autophosphorylation to activate its kinase activity following release from GRP78. Additionally, aberrant lipid metabolism of the ER membrane can directly activate PERK, demonstrating that lipid metabolism is a direct trigger of the UPR (22, 23). PERK phosphorylates the downstream eukaryotic translation initiator 2α (eIF2α), which reduces protein synthesis and consequently prevents the loading of nascent proteins into the ER lumen (5). Additionally, eIF2α can be phosphorylated in mammals independent of ER stress by three additional kinases, including protein kinase R (PKR), heme-regulated eIF2α kinase (HRI), and general control nonderepressible 2 kinase (GCN2). Phosphorylated eIF2α (p-eIF2α) tightly interacts with the guanine-nucleotide exchange factor (GEF) eIF2B. This process rapidly prevents the formation of the complex needed to load the 43S ribosome with methionine, thereby inhibiting the initiation of translation (24). However, several mRNAs can overcome this suppression, and they are selectively translated following an increase in p-eIF2α, including activating transcription factor 4 (ATF4). ATF4 plays essential roles in modulating the expression of genes involved in redox control, amino acid metabolism, autophagy, and protein synthesis and folding (25).

In addition, ATF4 promotes PPP1R15A expression, which subsequently induces the expression of DNA damage-inducible 34 (GADD34), a regulatory subunit of protein phosphatase 1 (PP1); this results in dephosphorylation of eIF2α, leading to restoration of mRNA translation (26). When ER stress is intense or persistent, ATF4 additionally promotes pro-apoptotic factors, such as C/EBP homologous protein (CHOP), and enhances oxidative stress, leading to cell death (27). PERK activation generally restores ER proteostasis; however, if the adaptive mechanism is overwhelmed, it triggers cell death cascades.

ATF6 is an ER-membrane-bound transcription factor that is a member of the basic leucine zipper protein (bZIP) transcription factor family, which is expressed in the ER and which senses various stress stimuli in different cell types (20). Upon ER stress, ATF6 is transferred to the Golgi apparatus, where it is cleaved by site-1 protease (S1P) and site-2 protease (S2P), leading to the release of its active cytosolic fragment (ATF6f) (28). ATF6f is then transported into the nuclear compartment, where it functions as a transcription factor and facilitates the transcription of various genes involved in protein folding, including ER protein chaperones; modulation of XBP1 mRNA levels; and ERAD (29, 30).

Overall, once activated, IRE1α, PERK, and ATF6 constitute sophisticated and coordinated mechanisms that are involved in easing the aberrant accumulation of misfolded or unfolded proteins within the ER lumen by upregulating the expression of ER chaperones that attenuate protein translation, inhibiting protein entry into the ER, and promoting the retrograde export of misfolded/unfolded proteins from the ER to the cytosol for proteasome-mediated degradation. However, if these adaptive mechanisms are overwhelmed, persistent UPR activation leads to cell death and inflammation.

## Role of Endoplasmic Reticulum Stress in Neuronal Death

Although limited neuronal death is a highly regulated and essential homeostatic mechanism for the maintenance of CNS functional development (31), pathological neuronal loss in mature CNS leads to irreversible functional decline of motor and cognitive function, ranging from acute CNS injuries, such as traumatic brain injury (TBI) and ischemic stroke, to chronic neurodegenerative diseases, such as Alzheimer’s disease (AD) and Parkinson’s disease (PD) (32). There are at least a dozen mechanisms for neuronal death resulting from various neurological diseases (32). Numerous modulatory mechanisms are involved in determining cell death, and ER stress and the UPR play essential roles in regulating mechanisms of programmed neuronal death, such as apoptosis, necroptosis, pyroptosis, ferroptosis and autophagy (33–35).

### Endoplasmic Reticulum Stress and Neuronal Apoptosis

If the adaptive mechanism fails to restore ER proteostasis, the UPR continues to activate and triggers three ER transducers and a series of downstream signaling pathways, leading to neuronal apoptosis. During the UPR, IRE1α-induced RIDD increases cell apoptosis by degrading mRNAs encoding growth-promoting proteins (18). Additionally, the IRE1α/TRAF2/c-Jun N-terminal kinase (JNK) pathway has been broadly reported to play an important role in regulating neuronal apoptosis in response to ER stress (36, 37). The phosphorylation of JNK potentiates BAX-dependent apoptosis by up-regulating the pro-apoptotic BCL-2 family member BIM and down-regulating the anti-apoptotic protein BCL-2 (38, 39). IRE1α is also reported to directly interact with two pro-apoptotic proteins, BCL-2 associated X protein (BAX) and BCL-2 antagonist/killer (BAK), to modulate IRE1α-mediated cell apoptosis (40). Finally, IRE1α-mediated phosphorylation has been reported to recruit caspase 12 (CASP 12), which is involved in regulating neuronal ER-specific apoptosis and cytotoxicity (41, 42). Persistent ER stress-induced PERK activation and concomitant downstream eIF2α/ATF4/CHOP activation play vital roles in promoting neuronal apoptotic death (43–45). CHOP, a transcription factor downstream of the PERK/ATF4 pathway, is thought to be essential to ER stress-induced apoptosis, as its depletion or inhibition attenuates tissue insults in response to ER stress (46–49). Downregulation of CHOP using siRNA significantly reduced neuronal apoptosis in a mouse model of intracerebral hemorrhage (ICH) (50). Importantly, CHOP is also reported to be a common downstream component at the convergence of the IRE1α, PERK, and ATF6 pathways (51). Moreover, CHOP has been demonstrated to bind to an element in the promoter of the gene encoding various pro-apoptotic members of the BCL-2 family (21). CHOP may also exacerbate ER stress-induced apoptosis by promoting the expression of GADD34, Tribbles-related protein 3 (TRB3), and endoplasmic reticulum oxidoreductin 1α (ERO1α) (52). Further, ATF6 is highly expressed in neurons and is well described to regulate neuronal apoptosis, primarily by upregulating the expression of CHOP (50, 53, 54).

### Endoplasmic Reticulum Stress and Neuronal Necroptosis

Different from apoptosis, necroptosis is a form of regulated necrotic cell death that occurs in a caspase-independent manner and is mediated by receptor-interacting protein kinase 1 (RIPK1), RIPK3, and mixed lineage kinase domain-like protein (MLKL). Recently, necroptosis has been shown to play an essential role in neuronal programmed death in various acute CNS injuries and neurodegenerative diseases, and occurs following disruption of the plasma membrane and cell lysis (55). In addition to the activation of various death receptors, intrinsic ER stress has been recently demonstrated to be an essential contributor to necroptosis. In both *in vitro* and *in vivo* models of cardiac ischemia-reperfusion, it was elucidated that RIPK3-induced necroptosis was primarily mediated by ER stress *via* the calcium overload/xanthine oxidase/reactive oxygen species/mitochondrial permeability transition pore opening pathway (56). Additionally, it was demonstrated that necroptosis induced by PFWRIRIRR-NH2 (PFR) was regulated by ER stress *via* cytoplasmic calcium overload/the mitochondrial reactive oxygen species axis (57). It has been demonstrated that necroptosis-induced by various ER stressors in fibroblasts is primarily mediated by tumor necrosis factor receptor 1 (TNFR1). Moreover, repression of RIPK1, RIPK3, or MLKL in these cells resulted in switching the form of ER stress-induced cell death from necroptosis to apoptosis (58). Additionally, one confounding study found that an inhibitor of PERK, GSK2606414, prevented necroptosis by blocking RIPK1 (59). Inhibiting ER stress by Tauroursodeoxycholic acid (TUDCA) prevents Necrostatin-1-induced necroptosis in NP cells (60). Additionally, another inhibitor of ER stress, 4-phenylbutyrate (4-PBA), has been shown to attenuate necroptosis of microglia (61). Consistently, an *in vitro* study investigating the effects of ER stress on neurotoxicity found that ER stress inhibitor 4-PBA and tangeretin significantly reduced JNK-medicated neuronal necroptosis (62). Furthermore, in a rat model of global cerebral ischemia, administration of the ER stress inhibitor salubrinal after cerebral ischemia significantly decreased the expression of necroptotic markers and reduced selective neuronal necroptosis (63).

### Endoplasmic Reticulum Stress and Neuronal Pyroptosis

Unlike apoptosis and necroptosis, pyroptosis is characterized by inflammasome-triggered, caspase 1- or caspase 11-mediated, and gasdermin-executed formation of plasma membrane pores and plasma membrane rupture. It is a rapid, inflammatory form of lytic programmed cell death (64). There is a growing appreciation for pyroptosis as a pivotal factor in neuronal death and neuroinflammation in neurological diseases (65). Recent studies have shown that ER stress is widely involved in multiple steps of pyroptosis induction (66). Recently, in a mouse model of acute hemorrhage stroke, ER stress was found to modulate neuronal pyroptosis by regulating the expression of IL-13 (67). IRE1α has been shown to play a pivotal role in NLRP3 inflammasome assembly, caspase-1 activation, and pro-IL-1β processing in monocytes and human peripheral blood mononuclear cells (PBMCs) (68). IRE1α activation was reported to induce NLRP3 inflammasome-mediated pyroptosis by increasing the expression of thioredoxin-interacting protein (TXNIP) (69, 70). TXNIP, an endogenous negative modulator of the antioxidant protein thioredoxins (TXNs), was recently reported to be closely associated with NLRP3 inflammasome activation (71). Consistent with this observation, in the cerebral venous sinus thrombosis (CVST) model, p-PERK and p-IRE1α were found to be expressed primarily in neurons; they contributed to neuronal pyroptosis by regulating TXNIP–NLRP3 inflammasome activation (72). In addition, recent research demonstrated that IRE1α could modulate NLRP1 inflammasome-mediated neuronal pyroptosis by regulating the expression of miR-125-b-2-3p (73). PERK inhibition has been reported to inhibit NLRP3 inflammasome activation by regulating the mitochondria-associated endoplasmic reticulum membrane (MAM)-induced calcium release (74). Further research showed that silencing PERK reduced the expression of TXNIP and NLRP3 in the tunicamycin-treated hepatocyte-derived AML 12 cell line (75). A recent study aimed at revealing the relationship between pyroptosis and ER stress found that the PERK inhibitor GSK2656157 inhibited GSDEME-mediated neuronal pyroptosis in neuronal cells exposed to methamphetamine (76). Cleaved ATF6 has been shown to be involved in NLRP3 inflammasome activation and pyroptosis in monocytes (77). Similarly, ATF6 has been reported to be associated with neuronal pyroptosis, where downregulation of ATF6 could reverse GSDEME-mediated pyroptosis in neuronal cells (76). Collectively, these studies imply that ER stress is involved in inflammasome-mediated neuronal pyroptosis.

### Endoplasmic Reticulum Stress and Neuronal Ferroptosis

Ferroptosis is an iron-dependent, non-apoptotic form of programmed cell death characterized by loss of activity of the lipid repair enzyme glutathione peroxidase 4 (GPX4) and concomitant accumulation of lipid reactive oxygen species (78). Although well described in cancer cells, ferroptosis has been reported to play pivotal roles in neurons in various neurological diseases (79). Recently, several studies have indicated a close correlation between ferroptosis and ER stress. First, inhibition of system Xc^−^ (a cell-surface cystine–glutamate antiporter) with several ferroptotic agents was shown to result in both eIF2α/ATF4 pathway activation and ferroptotic cell death (80, 81). Similarly, microassay studies showed that the ferroptosis activator glutathione S-transferase inhibitor artesunate (ART) increased the expression of ATF4-dependent genes such as CHOP and ASNS (82). Another recent study revealed that the PERK downstream protein ATF4 significantly increased the expression of system Xc^−^ and promoted tumor angiogenesis, which can be attenuated by pharmacological or genetic system Xc^−^ inhibition or ferroptotic agents such as sorafenib and erastin (83). Additionally, in both *in vivo* and *in vitro* models of glioma, the mechanism by which dihydroartemisinin exerts anticancer effects on glioma cells was found to be dependent on promoting ferroptosis *via* the PERK–ATF4–heat shock protein family A member 5 (HSPA5)–GPX4 pathway (84). Recently, emerging evidence revealed that nuclear factor erythroid 2-related factor 2 (NRF2) plays a key role in linking ferroptosis and ER stress. NRF2, a key orchestrator of the cellular antioxidant response, is directly regulated by PERK (85, 86). Growing evidence suggests that NRF2, as a transcription factor, plays a pivotal role in inducing the transcription of multiple genes involved in lipid peroxidation and regulators of ferroptosis, such as GPX4 and system Xc^−^ (87, 88). Finally, ROS, which play pivotal roles in lipid peroxidation-induced ferroptotic death, are also regulated by ER stress (89, 90). Overall, there was a close crosstalk between ER stress and ferroptosis. However, the precise molecular mechanism of the crosstalk between the three ER stress responses and neuronal ferroptosis signaling remains elusive, and further investigation is required to identify it.

### Endoplasmic Reticulum Stress and Neuronal Autophagy

Autophagy is the major intracellular degradation system by which cytoplasmic material is engulfed by autophagosomes and degraded upon autophagosome fusion with lysosomes (91). Adaptive autophagy in response to initial ER stress is usually considered a protective mechanism; however, the devastating ER stress can shift protective autophagy to compromised autophagy, leading to autophagic cell death (92). Dysfunction of autophagy flux determines neuronal death, which is involved in many neurological diseases in which ER stress plays an important regulatory role (33). In a mouse model of PD, it was first demonstrated that autophagy induced by mild ER stress inhibited neuronal death (93). Additionally, it was demonstrated in both *in vitro* and *in vivo* experimental models of diabetes that activation of the ER-stress-induced JNK signaling pathway may contribute to the induction of autophagy, which confers neuroprotection to ER-stress-associated neuronal damage (94). Furthermore, Japanese encephalitis virus (JEV)-induced autophagy has been shown to delay virus-induced neuronal death by activating ER stress and its downstream pathways (95). In another study, rapamycin-induced neuronal autophagy protected neurons from apoptotic death (96). However, excessive ER stress may lead to neuronal autophagic death. A recent study investigating the effects of pre-ischemia melatonin treatment on neuronal injury showed that the inhibition of ER stress-dependent autophagy significantly attenuated neuronal injury (97). Neuronal death promoted by fluoride-induced neurotoxicity has been demonstrated to primarily result from excessive ER stress-associated autophagic flux dysfunction (98). Additionally, in the Drosophila model of PD, IRE1α overexpression led to parkinsonian neurodegeneration through autophagy-dependent dopaminergic neuron loss (99). Similarly, hypercholesterolemia has been shown to activate the IRE1α/JNK signaling pathway in heart tissue, leading to autophagic cell death (100). Heme, which is released from hemorrhagic blood following intracerebral hemorrhage (ICH), was reported to increase autophagy-dependent neuronal death *via* ER stress (101). In glioblastoma cells, doxorubicin was reported to elicit persistent ER stress, thereby leading to autophagic and apoptotic cell death (102). A recent study revealed that knockout of ATF4 significantly inhibited compound loperamide-induced autophagic cell death in glioblastoma cells (103). Further investigation in this study showed that ATF4 is required for loperamide-induced reticulophagy (103), whereby autophagy degrades the ER itself, amplifying ER stress and cell death (104). Collectively, these observations indicate that ER stress-induced autophagy plays a dual role in determining neuronal fate; autophagy induced by mild ER stress plays a pro-adaptive role, while excessive or persistent ER stress conditions directly evoke a shift of autophagy toward its pro-death role, thereby leading to neuronal autophagic death.

## The Role of Endoplasmic Reticulum Stress in Innate Immune Response in the Central Nervous System

Emerging evidence indicates that neuroinflammation is a pathology common to various neurological disorders, including CNS injury and neurodegenerative diseases (105). Neuroinflammation is a complex immune response characterized by the activation of resident CNS glial cells (microglia and astrocytes), peripherally derived immune cells, and production of inflammatory cytokines and chemokines (106). As a key component of the CNS innate immunity, microglia- and astrocyte-mediated innate immune responses initially exert essential and functional roles in cellular debris clearance and neural tissue repair. However, under chronic pathological conditions, the innate immune response in the CNS gradually becomes an important contributor to the aggravation of blood–brain barrier (BBB) disruption, tissue damage, and neuronal death (107). ER stress not only regulates inflammatory responses in peripheral tissues but also plays a role in innate immune responses associated with neurological disorders (108).

### Endoplasmic Reticulum Stress and Inflammatory Pathways

**Figure 2:**
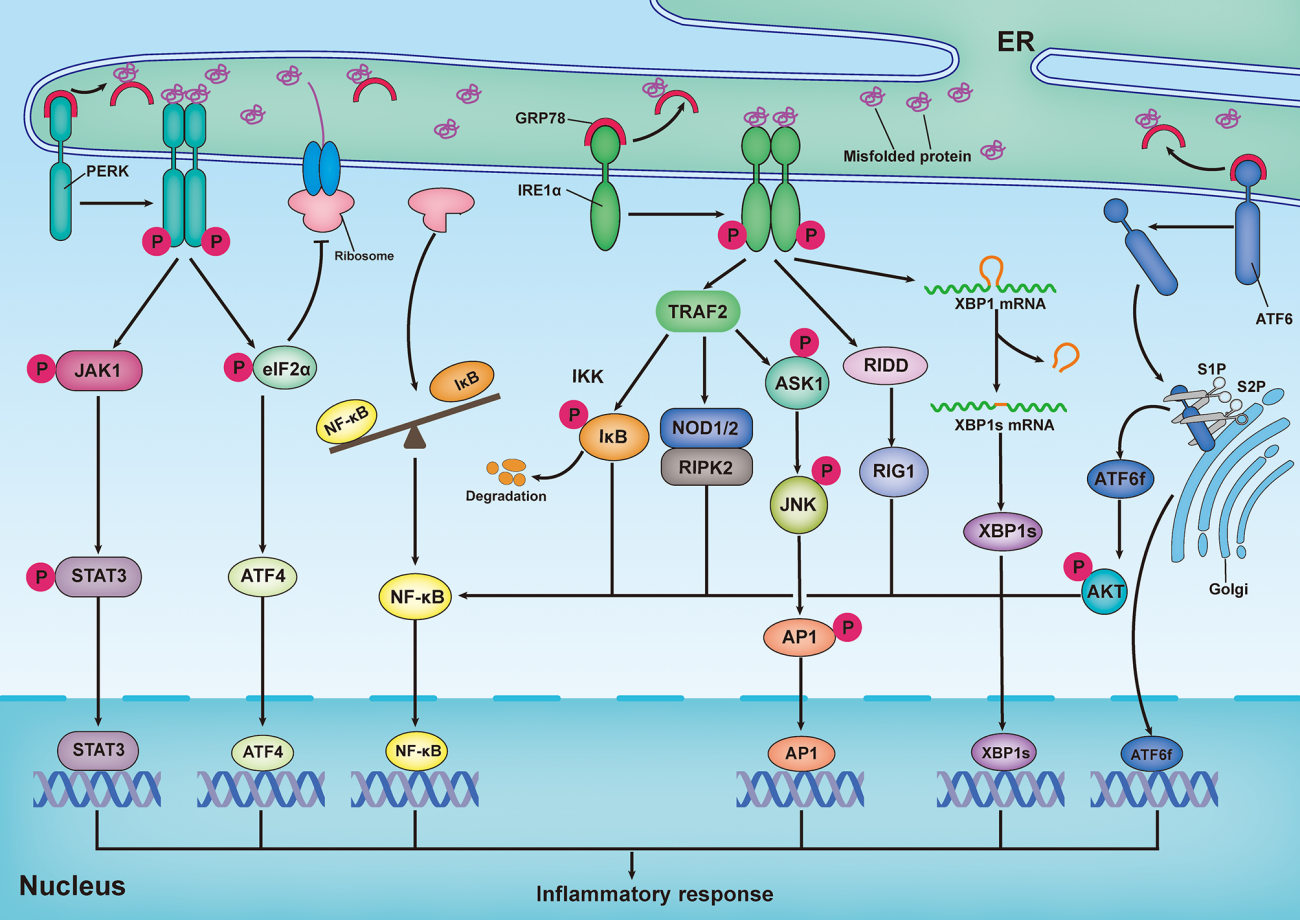
Inflammatory response induced by the unfolded protein response. Upon ER stress, PERK undergoes its dimerization and autophosphorylation to phosphorylate eukaryotic translation initiator 2α (eIF2α) and Janus kinase 1(JAK1), which respectively promote activating transcription factor 4 (ATF4) expression and signal transducer and activator of transcription 3 (STAT3) phosphorylation, thereby leading to inflammatory gene expression. In addition, translation attenuation by PERK-dependent phosphorylation of eIF2α results in decreased translation of both IκB and nuclear factor κB (NF-κB) but elevation of the proportion of NF-κB to IκB, owing to the shorter half-life of IκB, thereby promoting NF-κB-mediated inflammatory response. In response to ER stress, activation of inositol-requiring enzyme 1α (IRE1α) increase the expression of functionally active proteins of XBP1s, leading to inflammatory gene expression. During ER stress, interaction of IRE1α and TRAF2 can promote NF-κB-mediated inflammatory response by triggering IκB kinase (IKK)/κB pathway and nucleotide-binding oligomerization domain 1 and 2 (NOD1/2)/receptor-interacting serine/threonine-protein kinase 2 (RIPK2) pathways. In addition, both IRE1α-mediated IRE1α-dependent decay (RIDD)/retinoic-acid inducible gene 1 (RIG-1) pathway and IRE1α-induced expression of spliced x-box-binding protein 1 (XBP1s) are responsible for activating NF-κB. In addition to the activation of NF-κB, IRE1α-TRAF2 complex can also recruit apoptosis signal-regulating kinase 1 (ASK1) and subsequently activate JNK, thereby resulting in expression of pro-inflammatory genes by stimulating the bZIP transcription factor activator protein 1(AP-1). Upon ER stress, ATF6 is transported to Golgi, where it is cleaved by Site-1 protease (S1P) and Site-2 protease (S2P), releasing its active cytosolic fragment (ATF6f). ATF6f as a transcription factor, directly participate in regulating inflammatory response. Besides, ATF6f can also activate NF-κB by inducing the phosphorylation of the AKT.

ER stress has been implicated as playing a role in various inflammatory diseases by directly regulating the inflammatory pathways. Emerging findings reveal that there is a reciprocal regulation between ER stress and inflammatory responses (**Figure 2**). Previous studies have demonstrated that XBP1s, as a transcription factor, is implicated in pro-inflammatory gene expression (109, 110). ATF4 has been shown to directly interact with the IL-6 promoter to elicit a pro-inflammatory response (111). Additionally, in astrocytes, PERK directly interacts with Janus kinase 1 (JAK1) and promotes signal transducer and activator of transcription 3 (STAT3) phosphorylation in response to ER stress, thereby leading to inflammatory gene expression (112). Recently, the transcription factor ATF6 has been reported to increase the expression of various pro-inflammatory cytokines in response to persistent ER stress (113). Importantly, the NF-κB and mitogen-activated protein kinase (MAPK) family proteins JNK and p38 are the primary inflammatory signaling molecular players, which are directly initiated by ER stress (114). In turn, pro-inflammatory stimuli, such as ROS, Toll-like receptor (TLR) ligands, and cytokines, can aggravate ER stress and amplify inflammatory responses *via* a positive feedback loop (115).

NF-κB, a master transcription factor of pro-inflammatory pathways, plays a pivotal role in inflammation and immunity (116). During ER stress, the interaction of IRE1α and TRAF2 can lead to the recruitment of IκB kinase (IKK), as well as the phosphorylation and subsequent degradation of IκB (117). Subsequently, free NF-κB is transported into the nucleus, where it binds to κB sites in gene promoters and drives the expression of pro-inflammatory cytokines. Additionally, the IRE1α–TRAF2 complex can activate NF-κB by triggering the nucleotide-binding oligomerization domains 1 and 2 (NOD1/2)–receptor-interacting serine/threonine-protein kinase 2 (RIPK2) pathway (118, 119). Emerging evidence has suggested that the IRE1α/XBP1s pathway is also responsible for the activation of NF-κB (120–123). During the UPR, IRE1α-induced RIDD participates in promoting the production of retinoic acid-inducible gene 1 (RIG-1), which is responsible for activating NF-κB (124). Additionally, PERK and ATF6 have been reported to promote NF-κB activation; however, the mechanisms are different from those employed by IRE1α. In response to ER stress, activation of the PERK/eIF2α signaling pathway can result in decreased translation of both IκB and NF-κB. However, this results in an increase in NF-κB levels compared to IκB levels, because IκB has a shorter half-life than NF-κB, thereby promoting NF-κB-mediated inflammatory responses (125, 126). Moreover, the ATF6-mediated arm of the UPR has been demonstrated to activate NF-κB by inducing the phosphorylation of AKT (127, 128).

The MAPK family consists of stress-inducible kinases, including JNK, p38, MAPK, and extracellular signal-regulated kinase (ERK). These enzymes are serine-threonine protein kinases that mediate various biological processes, such as proliferation, apoptosis, and inflammation (129). In addition to the activation of NF-κB, the IRE1α-TRAF2 complex can recruit apoptosis signal-regulating kinase 1 (ASK1) and subsequently activate JNK, resulting in the expression of pro-inflammatory genes by stimulating the bZIP transcription factor activator protein 1(AP-1) (130, 131). Additionally, hyperactivation of IRE1α in response to inefficient XBP1 signaling can elicit inflammatory responses by activating JNK (132). In retinal Müller cells, hyperglycemia- and hypoxia-induced ER stress promotes activation of ATF4, which upregulates inflammatory genes by activation of hypoxia-inducible factor (HIF) and JNK, resulting in the retinal expression of inflammatory cytokine tumor necrosis factor (TNF-α) and VEGF (133). Furthermore, in an *in vitro* experimental model of ventilator-induced lung injury, inhibition of ER stress effectively prevented the inflammatory response by regulating NF-κB and the MAPK family (JNK, ERK, and p38) signaling pathways (134). Consistent with this observation, the inhibition of PERK by GSK2606414 dampened the receptor activator of NF-κB ligand (RANKL)-induced osteoclast differentiation through inhibition of MAPK and NF-κB (135).

### Endoplasmic Reticulum Stress and Microglia-Mediated Immune Response in the Central Nervous System

Microglia, resident macrophages of the CNS, play key roles in regulating brain development, maintaining neuronal networks, clearing cellular debris, and producing neurotrophic factors (136, 137). Microglia are always activated earlier than other types of glial cells under pathological conditions, such as acute brain injury and neurodegeneration; subsequently, they induce a broad spectrum of pro-inflammatory responses and neurotoxic factors, accelerating neuronal death and brain damage (105). However, microglia can also release anti-inflammatory cytokines and neurotrophic factors, which in turn produce neuroprotective effects (138). These distinct outcomes are determined according to the polarization state of the microglia; the M1 phenotype aggravates neuronal damage, and the M2 phenotype protects neurons from insults (139). The essential involvement of ER stress in the activation and polarization of microglia in neurological diseases has been widely reported.

In a study on the effects of mild ER stress on neuronal death, tunicamycin-induced mild ER stress was shown to significantly attenuate LPS-induced pro-inflammatory cytokine production in primary cultured microglia, which promoted the polarization of microglia from the M1 to M2 phenotype (140). In contrast, excessive ER stress triggers a series of pro-inflammatory signaling pathways in microglia, resulting in microglia-mediated neuroinflammation. Cocaine, one of the most commonly abused drugs, has been shown to promote microglia-mediated inflammatory responses (141). Further investigation revealed that ER stress elicited cocaine-induced autophagy, which in turn promoted microglial activation and the release of inflammatory cytokines (142). Consistent with this observation, another study showed that exposure of microglial cells to cocaine upregulated the expression of TLR2 through the PERK/ATF4 pathway, resulting in microglial activation and pro-inflammatory cytokine production (143). Furthermore, a recent study demonstrated that the protein tyrosine phosphatase 1 B (PTP1B) inhibitor sc-222227 effectively promotes microglial M1-to-M2 transformation and inhibits the release of microglia-mediated inflammatory cytokines following cerebral ischemia/reperfusion (IR) injury by modulating the ER stress–autophagy axis *via* PERK signaling in microglia (144). These observations collectively indicate that the ER stress-autophagy axis plays an essential role in modulating microglia-mediated neuroinflammation. Additionally, another study that explored the association between the extent of UPR signaling and type I interferons (IFN) showed that the impairment of proteasome activity in microglia induced an IFNβ1 response and concomitant IL-6 secretion in an IRE1α-dependent manner. In this case, inhibition of IRE1α endoribonuclease activity attenuated Tank-binding kinase 1-mediated type I IFN activation (145). Interestingly, this study further showed that both PERK/CHOP and IRE1α/XBP1s are involved in modulating IL-6 secretion in microglia (145). Furthermore, the eIF2α/ATF4 signaling pathway has been reported to be involved in modulating microglial activation and M1/M2 polarization, which in turn regulates microglia-mediated generation of inflammatory cytokines (146–149). Recently, MAP kinase phosphatase 1 (MKP1), an antiapoptotic protein, has been shown to inhibit microglial activation by modulating ER stress and mitochondrial function (150). Additionally, a previous study that explored the role of ATF6α in experimental autoimmune encephalomyelitis (EAE) mice found that ATF6α deficiency effectively inhibited microglial activation and pro-inflammatory cytokines by activating NF-κB signaling, thereby ameliorating demyelination and clinical symptoms (151).

Collectively, these findings suggest that ER stress plays a pivotal role in regulating microglial functions under both physiological and pathological conditions because of the key signaling molecules in its downstream signaling pathway. Indeed, in response to insult and injury, cellular ER stress, through its downstream signaling pathways, directly participates in modulating microglia-mediated neuroinflammation. However, the mechanism by which this occurs has only been partly elucidated; further investigation needs to conduct regarding the interplay between ER stress; microglial activation; and neurological diseases such as PD, AD, and TBI.

### Endoplasmic Reticulum Stress and Astrocyte-Mediated Immune Response in the Central Nervous System

Astrocytes are the most abundant glial cells; they perform essential functions in the CNS physiology, including synaptogenesis, neurotransmission, BBB formation, and metabolic regulation (152). Meanwhile, reactive astrocytes can also participate in CNS inflammatory responses and exacerbate neuroinflammation by interacting with other immune cells. Indeed, reactive astrocytes responding to various insults or injuries can directly secrete a series of pro-inflammatory cytokines, such as IL-1β and TNF-α, along with increased generation of ROS, thereby playing an essential role in neurological diseases and neurological outcomes (153, 154). Astrocytes undergo molecular and morphological changes in response to various stresses, a process known as astrogliosis (155). Astrogliosis is characterized by increased levels of glial fibrillary acidic protein (GFAP), proliferation, and hypertrophy. Recently, single-cell RNA sequencing (scRNA-seq) has been used for the detection of increased UPR signaling in expanded subpopulations of astrocytes during EAE (156). Astrocytes express all three trans-ER membrane stress sensors (IRE1α, PERK, and ATF6) and specifically express old astrocyte specifically induced substance (OASIS). Accumulating evidence suggests that aberrant ER stress in astrocytes plays a pathological role in neuroinflammation in various neurological diseases (157).

In addition to its implication in microglial activation, the ER stress-autophagy axis has been reported to play an essential role in astrocyte activation and concomitant inflammatory responses (158, 159). Preconditioning of cells with mesencephalic astrocyte-derived neurotrophic factor (MANF) and conserved dopamine neurotrophic factor (CDNF) has been shown to effectively inhibit ER stress and alleviate cell damage and inflammatory cytokine production in rat primary astrocytes (160, 161). Additionally, preconditioning of astrocytes with progesterone effectively protects them from amyloid β (Aβ)-induced inflammation by suppressing ER stress activation (162). Selenoprotein S, an ER-resident protein, attenuates pro-inflammatory responses by reducing ER stress in astrocytes (163). Accumulating evidence indicates that the PERK-mediated arm of the UPR in astrocytes is linked to the induction of inflammatory responses. Moreover, recent evidence has indicated that genetic haploinsufficiency or inhibition of PERK in primary astrocytes inhibits ER stress-associated inflammation (IL-6, CCL2, and CCL20 production) at both the mRNA and protein levels (164). Additionally, the interplay between the PERK-mediated arm of the UPR and JAK/STAT-signaling-dependent inflammation has been well described in astrocytes (112, 164–166). The JAK1/STAT3 pathway regulates the expression of various inflammatory genes, as well as immunological functions (167). PERK signaling-induced activation of JAK1/STAT3 results in the generation of various cytokines, such as IL-6 and oncostatin M (OSM).

Additionally, free IL-6 can bind to its cell membrane receptor and further activate JAK1/STAT3 (168). Thus, these free IL-6 cytokines may synergize with ER stress in astrocytes, amplifying inflammation (112). Recently, a study investigating the role of IRE1α in a mouse model of AD found that genetic ablation of the RNase domain of IRE1α significantly reduced amyloid deposition and astrogliosis (169). Furthermore, another study aimed at identifying signaling pathways involved in pathogenic neuroinflammation in multiple sclerosis (MS), revealed that IRE1α/XBP1 signaling in astrocytes is a driver of genomic programs and that it modulates astrocyte pro-inflammatory activities (170).

Collectively, these *in vivo* and *in vitro* findings revealed that ER stress plays an essential role in modulating astrocyte activation and astrocyte-driven inflammatory activities in the CNS. However, studies on the implications of ATF6 and OASIS in astrocyte activation during various neurological diseases remain undefined, and further investigations are required in this regard.

## The Role of Endoplasmic Reticulum Stress in Neurological Diseases

The aberrant accumulation of misfolded/unfolded proteins inside the ER lumen activates ER stress responses, which subsequently trigger sophisticated and coordinated UPRs, thereby restoring homeostasis and normal ER function (**Figure 1**). Indeed, the adaptive UPR can enhance the adaptive capacity of the ER and promote cell resistance to external stimuli by altering the transcriptome and proteome (171). However, the intense and devastating ER stress that occurs in acute CNS injury and the prolonged ER stress that develops in neurodegenerative disorders, are considered to disrupt the protective mechanisms of the UPR, resulting in the activation of neuronal death and inflammatory responses in the CNS. In the following sections, we briefly generalize the roles of ER stress in multiple neurological diseases, ranging from acute CNS injuries to neurodegenerative diseases (**Table 1**).

**Table 1:** Functional impact of ER stress in neurological diseases.

Disease	Model	Intervention	Effects	Refs
ICH	Autologous blood-induced ICH	TUDCA (ER stress inhibitor)	Neuroprotection	(172)
		siRNA CHOP	Inhibited ER stress-associated neuronal apoptosis; alleviated neurological deficits	(50)
	Collagenase-induced ICH	TUDCA (ER stress inhibitor)	Alleviated neurological deficits	(67)
		PERK inhibitor GSK2606414	Inhibited ER stress-associated neuronal apoptosis	(173)
IS	OGD/R	PERK inhibitor GSK2606414	Neuroprotection; attenuated the ER stress-associated inflammation	(144) (174)
		siRNA ATF4	Promoted primary neuronal apoptosis	(175)
		Overexpression of IRE1α	Promoted ER stress-associated primary neuronal apoptosis	(176)
		Overexpression of XBP1	Inhibited primary neuronal cell death	(177)
	OGD/R; tMCAO	4-PBA (ER stress inhibitor)	Inhibited ER stress-associated neuronal apoptosis and inflammation	(178–183)
	tMCAO	TUDCA (ER stress inhibitor)	Inhibited ER stress-associated neuronal apoptosis; alleviated neurological deficits	(184)
		PERK inhibitor GSK2606414	Inhibited ER stress-associated neuronal apoptosis	(185)
		PERK cKO	Aggravated neurological deficits	(186)
		Overexpression of ATF4	Neuroprotection; alleviated neurological deficits	(187)
		ATF6 KI	Neuroprotection; alleviated neurological deficits	(188)
		147 (ATF6 activator)	Neuroprotection; alleviated neurological deficits	(189)
TBI	BOE	Salubrinal (eIF2α dephosphorylation inhibitor)	Inhibited ER stress-associated neuronal apoptosis and inflammation; alleviated impulsive-like behavior	(190–192)
	LFP	salubrinal (eIF2α dephosphorylation inhibitor)	Neuroprotection; attenuated the ER stress-associated neuronal apoptosis	(193)
	CCI	TUDCA (ER stress inhibitor)	Inhibited ER stress-associated neuronal apoptosis; alleviated neurological deficits	(194)
		Salubrinal (eIF2α dephosphorylation inhibitor)	Inhibited ER stress-associated neuronal apoptosis and inflammation; alleviated neurological deficits	(195, 196)
		Low dose guanabenz (an activator of eIF2α phosphorylation)	Inhibited ER stress-associated neuronal cell death; alleviated neurological deficits	(197, 198)
		PERK inhibitor GSK2656157	Inhibited ER stress-associated neuronal cell inflammation; alleviated memory deficits	(199, 200)
		CHOP KO	Reduced newborn neurons loss; improved cognitive come	(198)
AD	APP/PS1 mice	PERK cKO	Improved synaptic plasticity and spatial memory and LTP	(201) (202)
		siRNA ATF4	Neuroprotection	(203)
		overexpression of XBP1	Improved memory deficits; restored spine density and synaptic plasticity	(204)
		Overexpression of ATF6	Protected retention of spatial memory	(205)
	5XFAD mice	PERK+/-	Restored memory deficits and cholinergic neurodegeneration	(206)
		IRE1α cKO	Improved synaptic function and LTP; restored learning and memory functions	(169)
	Tau Tg mice	PERK inhibitor GSK2606414	Reduced brain atrophy and abrogated the appearance of clinical signs	(207)
		PERK inhibitors trazodone and dibenzoylmethan	Restored memory impairment, abrogated neurological signs, prevented neurodegeneration, and prolonged survival	(208)
	2 × Tg mice.	PERK inhibitor ECH	Ameliorated memory deficit	(209)
PD	α-synuclein Tg mice	Salubrinal (eIF2α dephosphorylation inhibitor)	Attenuated the progressive motor deficits	(210)
		PERK inhibitor GSK2606414	Attenuated DA neuronal cell death; improved motor performance	(211)
	Pink1/parkin mutant flies	PERK inhibitor GSK2606414	Neuroprotection	(212)
	Neurotoxins treated primary neurons	eIF2α inhibitor C16	Attenuated neuronal cell death	(213)
	Neurotoxins treated rat	overexpression of XBP1	inhibited DA neuronal degeneration	(214)
	Neurotoxins treated mice	overexpression of XBP1	Attenuated DA neuronal cell death	(215)
		ATF6 KO	Accelerated neuronal cell death	(216, 217)
		CHOP KO	Neuroprotection	(47)
		overexpression of XBP1	Attenuated DA neuronal cell death	(218)
MS	EAE mice	temporally controlled activation of PERK	Reduced oligodendrocytes loss, demyelination, and axonal degeneration	(219)
			Promoted cell survival and remyelination	(220)
			prevented neuron loss	(221)
		ATF6α KO	increased oligodendrocyte death and myelin loss	(222)
		PERK KO	Drive neuroinflammation	(112)
		OL-PERK ko/ko	Increased oligodendrocytes loss, demyelination, and axonal degeneration	(223)
	EAE/optic neuritis mice	CHOP deletion	Promote RGC soma and axon survival	(224)
		overexpression of XBP1	Promote RGC soma and axon survival	(224)
HD	Htt 150Q cells	overexpression of GRP78	reduced formation of mHtt aggregates; prevented cell death	(225)
	polyQ-expanded Htt cells	PERK inhibitor A4	Reduced mHtt cytotoxicity	(226)
	Htt 120Q cells	Salubrinal (eIF2α dephosphorylation inhibitor)	Prevented cell death	(227)
		IRE1α inhibitor Usp14	Protect against cell degeneration and cell death	(228)
	SH-SY5Y cells	shRNA IRE1α	Reduced neuronal toxicity	(229)
	AAV-Htt588Q95-mRFP mice	overexpression of XBP1	reduced the accumulation of mHtt inclusion	(230)
	YAC128 HD Mice	XBP1 cKO	Decreased the levels of mHtt	(231)
		shRNA IRE1α	Reduced the aggregation of pathological polyQ79-EGFP peptides	(231)
		shRNA XBP1	Reduced the aggregation of pathological polyQ79-EGFP peptides	(231)
ALS	SOD1 mutant mice	Salubrinal (eIF2α dephosphorylation inhibitor)	Ameliorated disease severity and delay progression	(232)
	SOD1 mutant neuro2a cells	Salubrinal (eIF2α dephosphorylation inhibitor)	Reduced cell death	(233)
	SOD1 mutant mice	PERK+/-	Exhibited an earlier disease onset, reduced lifespans, and earlier neuropathological alterations in spinal cord	(234)
		GADD34 dysfunction	Exhibited a delayed disease onset, delayed early phase of disease and prolonged lifespans	(235)
		shRNA GADD34	Ameliorated disease severity and prolonged lifespans	(236)
		Guanabenz(eIF2α dephosphorylation inhibitor)	Ameliorate disease severity with a delay in the onset and prolongation of the early phase of disease and survival	(237)
			Exhibited delayed onset of disease symptoms, prolonged lifespan and improved motor performance	(238)
		PERK inhibitor ISRIB	Reduced neuronal death	(239)
	mutant TDP-43 mice	Guanabenz(eIF2α dephosphorylation inhibitor)	Ameliorate motor deficits and axon defects	(240)
		Salubrinal (eIF2α dephosphorylation inhibitor)	Ameliorate motor deficits and axon defects	(240)
		PERK inhibitor GSK2606414	Inhibited ER stress-associated TDP-43 toxicity	(241)

ICH, intracerebral hemorrhage; IS, ischemic stroke; TBI, traumatic brain injury; AD, Alzheimer disease; PD, Parkinson’s disease; TUDCA, Tauroursodeoxycholic acid; tMCAO, transient middle cerebral artery occlusion; CCI, controlled cortical impact; BOE, blast overpressure exposure; LFP, lateral fluid-percussion; OGD/R, oxygen and glucose deprivation followed by reoxygenation; 5XFAD, five familial Alzheimer disease; KO, knockout; cKO, conditional knockout; KI, knockin; LTP, long-term potentiation; Tg, transgenic; DA, dopaminergic; EAE, experimental autoimmune encephalomyelitis; SOD, Cu, Zn-superoxide dismutase; TDP-43,TAR DNA Binding Protein43;Htt 150Q cells, mutant huntingtin containing 150Q cells: Usp14, Ubiquitin-specific protease-14.

### The Role of Endoplasmic Reticulum Stress in Intracerebral Hemorrhage

Spontaneous ICH is defined as bleeding within the brain parenchyma caused by a ruptured brain aneurysm. ICH is the most lethal subtype of stroke, with high mortality, morbidity, and recurrence rates. Currently, there is no effective treatment for improving the functional outcomes in patients with ICH (242). Primary brain injury refers to the formation and expansion of hematomas within the first few hours after ICH, which results in mass effects and increased intracranial pressure that can lead to herniation and death of patients. Secondary brain injury is complex; however, it primarily results from hematoma-induced edema, inflammatory activities, oxidative stress, and toxic biochemical effects (243, 244). Recently, ER stress has been considered as one of the molecular mechanisms involved in ICH-induced secondary brain injury (33).

The first evidence of activated ER stress in ICH was reported in patients with ICH with pathogenic mutations, two of the three putative mutations being COL4A2**^E1123G^** and COL4A2**^Q1150K^** (245). In a rat model of ICH, ER stress was activated after ICH, and inhibition of ER stress by TUDCA exerted neuroprotective effects in the brain (172). Importantly, it was indicated in a mouse model of ICH that TUDCA-induced inhibition of ER stress significantly alleviated neurological deficits by preventing neuronal pyroptosis *via* decreasing IL-13 expression (67). Additionally, in both *in vivo* and *in vitro* experimental models of ICH, the PERK inhibitor GSK2606414 effectively inhibited ER-stress-associated neuronal apoptosis following ICH, whereas the inhibitory effect was abolished in mice or cells receiving the eIF2α dephosphorylation inhibitor salubrinal (173). A study that explored the neuroprotective effects of melatonin in ICH showed that siRNA-induced inhibition of CHOP signaling effectively attenuated the pro-apoptotic effects of ATF6 induced by ICH (50). Heme, which is released from hemoglobin or other heme proteins following ICH, has been reported to increase autophagic neuron death by directly activating ER stress (101, 246). Moreover, oligodendrocyte apoptosis, which is associated with ICH-induced demyelination, has been reported to be mediated *via* ER stress (247).

### The Role of Endoplasmic Reticulum Stress in Ischemic Stroke

Stroke is the second leading cause of death and a leading cause of disability worldwide (248). Ischemia stroke, defined as arterial occlusion, represents approximately 71% of all strokes globally (249). Currently, the main practices to recanalize blood flow in acute ischemic stroke include intravenous tissue plasminogen activator (t-PA) and endovascular thrombectomy for large-vessel occlusion. Nevertheless, cerebral-ischemia-induced hypoxia and subsequent reperfusion-induced damage impair proteostasis and subsequently induce ER stress, leading to further devastating brain injury (250).

In a 2002 study, Ito et al. first reported that the expression of the ER stress markers GRP78 and IRE1α signaling molecules was increased in the ischemic rat brain (251). Importantly, in the same year, in mice, administration of the ER stress inhibitor TUDCA after transient middle cerebral artery occlusion (tMCAO) was shown to reduce the number of apoptotic cells and infarct size, as well as improve neurological outcomes (184). Additionally, in cerebral ischemic mice, PERK signaling molecules were found to be strongly increased (252). This study further indicated that tPA directly binds to cell surface GRP78, which, in turn, triggers a signal that attenuates ER stress overactivation in neurons, providing neuroprotection in ischemic mice (252). Sodium 4-phenylbutyrate (4-PBA sodium), a chemical chaperone that reverses misfolded proteins, was reported for the first time to reduce ER-stress-induced neuronal death in mice subjected to cerebral ischemic injury (178). Subsequently, emerging evidence has considered 4-PBA as an inhibitor of ER stress, and *in vitro* and *in vivo* studies in ischemic stroke models have shown that inhibition of ER stress by 4-PBA could alleviate neuronal death, endothelial cell damage, and proinflammatory responses (179–183). Recently, it was reported that Hes1 knockdown significantly aggravated neuronal apoptosis by activating the PERK/eIF2α/ATF4/CHOP signaling pathway after tMCAO, whereas this effect was effectively counteracted in mice receiving the PERK inhibitor GSK2606414 (185). Consistent with this result, a previous study demonstrated that inhibition of PERK by GSK2606414 could significantly downregulate the expression of proinflammatory cytokines in primary microglia induced by oxygen and glucose deprivation followed by reoxygenation (OGD/R) (144). Another *in vitro* study demonstrated that preconditioning of murine cortical glial cells with GSK2606414 protected them from OGD/R-induced cell damage (174). However, a recent study demonstrated that deletion of PERK in neurons resulted in a larger infarct volume and worse neurological function scores in mice subjected to MCAO compared to that in control mice (186). A study that investigated the function of ATF4 and its underlying mechanism in cerebral ischemia/reperfusion (I/R) injury showed that overexpression of ATF4 alleviated rat cerebral I/R injury by modulating the mitophagy–NLRP3 inflammasome axis (187), while ATF4 knockdown by siRNA induced the opposite effect. Similarly, in an *in vitro* stroke model of OGD, silencing of *ATF4* reversed tunicamycin-and thapsigargin-induced moderate ER stress and subsequent mitophagy induction, resulting in an increased apoptotic primary neuron cell ratio (175). In an *in vitro* model of ischemic strokes, overexpression of IRE1α enhanced ER stress and concomitant primary neuron death (176). However, in an *in vitro* model of I/R, adenovirus-induced XBP1 overexpression inhibited rat primary hippocampal neuron death induced by OGD/R stress (177). In a 2017 study, Yu et al. generated a conditional and inducible short form of ATF6 knock-in (ATF6-KI) mouse, in which sATF6 was primarily expressed in the neurons of the hippocampus and cortex. Preconditioning of the ATF6-KI mice with tamoxifen after tMCAO resulted in a reduced infarct volume and better neurological outcome compared to the results in the control mice (188). Recently, the administration of compound 147, an activator of ATF6, in mice resulted in significantly decreased brain infarct sizes and alleviated neurological deficits after I/R (189).

Collectively, these findings indicate the dual roles of ER stress in ischemic stroke, in which proteostasis disturbance induces ER stress and overactivation of the ER stressors PERK and IRE1α, resulting in harmful effects in ischemic stroke. In contrast, overexpression of downstream molecules, such as ATF4 and XBP1, or upregulation of ATF6, provides neuroprotection in ischemic stroke.

### The Role of Endoplasmic Reticulum Stress in Traumatic Brain Injury

TBI is a significant cause of mortality and long-term disability worldwide; it contributes to the economic burden on society and the low quality of life of affected individuals (253). As TBI is a highly heterogeneous injury with a complicated pathogenesis, its treatment has always been challenging. TBI is considered a “biphasic injury” characterized by an initial primary brain injury and delayed secondary brain injury. The primary brain injury causes irreversible brain damage as a consequence of mechanical injury, whereas the secondary brain injury results in further damage due to injurious biochemical cascades (254). Importantly, devastating ER stress is considered a primary contributor to the regulation of the inflammatory response and neural death in secondary brain injury.

In 1995, two studies reported significant elevation of the ER-resident protein GRP78 in the rat brain following TBI (255, 256). GRP78 is currently considered a marker of ER stress, and alterations in GRP78 expression can modulate cell apoptotic death following brain injury (257). ER stress and concomitant UPR have been well established to be involved in brain injury (258). TUDCA, as a potent inhibitor, has been reported to effectively attenuate ER stress and subsequent neuronal apoptotic death in mice subjected to TBI, thereby improving neurological function (194). In a blast injury model of TBI, the number of apoptotic neurons and expression of apoptotic proteins were found to be significantly reduced in mice receiving the ER stress inhibitor salubrinal, also known as the eIF2α dephosphorylation inhibitor (190). Consistent with this observation, multiple studies reported that salubrinal confers neuroprotection by attenuating ER-stress-associated neuronal cell death and neuroinflammation after TBI (191–193, 195, 196, 259). Concomitantly, the administration of low-dose guanabenz (an activator of eIF2α phosphorylation) to mice after TBI could reduce cortical and hippocampal tissue and neuron loss, which in turn could improve motor and cognitive functions (197, 198). These findings suggest that prolongation of eIF2α phosphorylation in acute TBI models is neuroprotective. In a mouse model of TBI, GSK2656157-treated mice exhibited increased expression of postsynaptic density 95 (PSD95), as well as better spine density and memory function compared to control mice after TBI (199). Additionally, GSK2656157 treatment of mice post injury promoted microglial phenotypic transformation from a pro-inflammatory M1-phenotype to an anti-inflammatory M2-phenotype and inhibited TBI-induced Th1 T cell infiltration, thereby alleviating white matter damage and symptoms of anxiety and depression (200). A recent study revealed that mice with a knockout of proapoptotic transcription factor CHOP exhibited reduced hippocampal newborn neuron loss and improved performance in context fear discrimination when compared to control mice following TBI (198). Finally, emerging evidence supports the claim that docosahexaenoic acid (DHA) is an effective inhibitor of ER stress (260). Moreover, some findings suggest that DHA administration to mice after TBI could significantly inhibit ER-stress-associated neuron damage and neuroinflammation and improve neurological function (261–263).

Collectively, these studies indicate that ER stress is implicated in secondary brain injury after TBI and that targeting ER stress responses may be a mechanism to attenuate neuronal cell death and inflammatory responses, as well as improve neurological function in TBI.

### The Role of Endoplasmic Reticulum Stress in Alzheimer’s Disease

AD is the most common cause of dementia and is characterized by progressive cognitive dysfunction and memory loss (264). AD affects more than 50 million people worldwide, and there are currently no effective drugs that can slow the progression of AD. The pathological hallmarks of AD include extracellular parenchymal deposition of Aβ, intracellular tau-containing neurofibrillary tangles (NFTs), and subsequent neuronal death and synaptic loss (265, 266). During AD, the continuous generation of Aβ and phosphorylated tau (p-tau) causes a disturbance of ER calcium homeostasis and aberrant protein folding in the ER, finally eliciting intracellular induction of ER stress and concomitant UPR activation (267). Additionally, a previous study showed the co-localization of p-PERK with p-tau in the hippocampus of aged TgTau**^P301L^** mice and confirmed a crosstalk between ER stress and hyperphosphorylation of tau in primary cultures of cortical neurons (268). Tau proteins directly impair the ERAD pathway, resulting in the accumulation of misfolded proteins within the ER lumen (269). Additionally, Aβ oligomers can directly interact with neuronal *N*-methyl-d-aspartate receptors (NMDARs), provoking downstream ER-stress-mediated cell death, synaptic depression, and spine elimination (270). An increasing number of studies have explored the potential therapeutic targets of TBI by unraveling the underlying mechanism of the correlation between ER stress and the pathogenesis of AD.

Several studies have reported the involvement of ER stress in different human brain regions in patients with AD (271–276). PERK and eIF2α are closely associated with the pathological alteration of neurons in patients with AD (277). Cognitive deficits and memory loss in patients with AD are reported to be associated with the expression of p-eIF2α in the brain (278, 279). PERK insufficiency has been shown to effectively inhibit β-secretase enzyme BACE1 expression and concomitant Aβ peptides and plaque burden, resulting in the restoration of memory deficits and cholinergic neurodegeneration in five familial AD (5XFAD) mouse models (206). Moreover, in a mouse model of AD, inhibition of eIF2α phosphorylation by conditional deletion of PERK effectively decreased amyloidogenesis and restored normal expression of plasticity-related proteins, thereby improving synaptic plasticity and spatial memory in AD mice (201). Consistently targeting PERK expression in the brain in a mouse model of AD demonstrated that deletion of PERK improved memory impairment and long-term potentiation (LTP) (202). Furthermore, the pharmacological inhibition of PERK by GSK2606414 restored the cognitive deficits in AD mice, which was associated with improved hippocampal metabotropic glutamate receptor (mGluR)-long-term depression (LTD) impairments (202). In a mouse model of frontotemporal dementia, GSK2606414 treatment of mice effectively restored protein synthesis rates and reduced neuronal loss in the brain, further reducing brain atrophy and abrogating the appearance of clinical signs (207). Echinacoside (ECH), another inhibitor of PERK, was reported to dramatically suppress Aβ generation and accumulation by inhibiting the translation of BACE1, ameliorating memory deficits in AD mice (209). A recent study demonstrated that treatment of AD mice with two PERK inhibitors, trazodone and dibenzoylmethane, effectively restored memory impairment, abrogated neurological signs, prevented neurodegeneration, and prolonged survival (208). Additionally, both eIF2α kinase GCN2 (201) and double-stranded RNA-dependent kinase (PKR) (280, 281) have also been reported to be involved in the regulation of memory impairment in *in vivo* models of AD. In an *in vitro* AD model, inhibition of eIF2α dephosphorylation by salubrinal effectively increased the translation of BACE1 and production of Aβ in primary neurons (275). Moreover, the expression of ATF4 at the mRNA and protein levels is significantly upregulated in the brains of patients with AD. Studies in mouse models and cell culture have demonstrated that axonally synthesized ATF4 is required to transmit neurodegenerative signals through cell-nonautonomous mechanisms (203). The correlation between the pathology of AD and the activation of IRE1α was detected in the brains of patients with AD (169). Genetic ablation of the RNase domain of IRE1α reduced amyloid deposition and astrocyte activation in the cortical and hippocampal areas of AD mice. Moreover, IRE1α deletion in the brains of AD mice significantly improved synaptic function and LTP, thereby restoring learning and memory functions (169). Furthermore, the transcription factor XBP1 is considered an essential contributor to the progression of learning and memory deficits. In line with this, mice with XBP1 conditional knockout showed significant learning and memory deficits, whereas mice with XBP1 overexpression exhibited improved LTP and synaptic transmission, both of which are associated with better learning and memory functions (282).

This concept has been applied to a mouse model of AD, in which virus-mediated delivery of the active spliced transcription factor XBP1s in the hippocampus improved memory deficits and restored spine density and synaptic plasticity (204). Studies in fly models have also demonstrated that XBP1 overexpression in neurons protects against neurotoxicity induced by Aβ and tau (283, 284). The role of ATF6 in AD has not been reported until recently, when Du et al. found a reduction in ATF6 expression in the brains of AD mice (205). This study demonstrated, both *in vivo* and *in vitro*, that ATF6 overexpression reduced Aβ production by inhibiting amyloid precursor protein (APP) expression, downregulated the promoter activity and expression of BACE1, and protected the retention of spatial memory in AD mice.

Collectively, numerous studies have demonstrated the involvement of ER stress in the pathogenesis of AD. Pharmacological or genetic targeting of ER stress may represent a strategy for attenuation of the continuous generation of Aβ- and p-tau-associated neuronal cell death and neuroinflammation, which, in turn, may improve memory deficits and synaptic plasticity in AD. However, most studies have focused on the underlying mechanism of PERK in AD, and further investigation is required to clarify the additional roles of IRE1α and ATF6 in AD.

### The Role of Endoplasmic Reticulum Stress in Parkinson’s Disease

PD is a chronic and progressive neurodegenerative disorder characterized by classic motor symptoms, such as tremor and bradykinesia, and non-motor manifestations, such as rapid eye movement sleep disorder, anosmia, constipation, and depression) (285). Pathologically, the symptoms of PD are accompanied by a profound loss of dopaminergic (DA) neurons in the substantia nigra pars compacta (SNpc). Dopaminergic neuron degeneration is associated with the appearance of Lewy bodies, which are predominantly composed of misfolded and/or mutated α-synuclein proteins (286). Emerging evidence supports the concept that the aberrant accumulation of misfolded and/or mutated proteins, such as α-synuclein, contributes to persistent ER stress and concomitant UPR activation, thereby resulting in dopaminergic neuronal death and degeneration, which is associated with PD (287).

Postmortem evidence has shown that upregulation of ER stress markers, such as in GRP78, p-PERK, and p-eIF2α, is detected in neuromelanin-containing DA neurons in the SNpc in the brains of humans with PD (211, 267, 288, 289). Furthermore, the colocalization of p-PERK and α-synuclein was detected in the SNpc of PD human brains (290). Similarly, α-synuclein was found to be broadly expressed in ER/microsomes fractions in the brains of both humans and mice with PD (210). Moreover, α-synuclein has been shown to directly bind to GRP78, and α-synuclein overexpression increases the sensitivity of neuronal cells to ER-stress-induced toxicity. These findings indicate that α-synuclein directly regulates ER-stress-associated neuronal death (210). Several studies have consistently demonstrated that α-synuclein proteins regulate ER stress by directly interacting with GRP78 (291, 292). Several *in vivo* and *in vitro* experimental studies have revealed that inhibition of ER stress exerts protective effects in PD physiopathology.

Salubrinal, an inhibitor of ER stress, has been reported to attenuate progressive motor deficits without protecting against neuronal death in an AAV-rat model of DA neurodegeneration (210). Subsequent studies have reported the potential pharmacological use of salubrinal in PD pathology (293–295). Recently, the targeting of PERK inhibition in a mouse model of PD indicated that the PERK inhibitor GSK2606414 protected DA neurons in the SNpc against cell death induced by the PD neurotoxin 6-hydroxydopamine (6-OHDA), thereby improving the motor performance of PD mice (211). Similarly, pharmacological inhibition of PERK with GSK2606414 was neuroprotective in the pink1/parkin model of PD (212).

The PD neurotoxins 1-methyl-4-phenyl-pyridinium (MPP**^+^**) and 6-OHDA have been shown to promote DA neuronal cell death in an ATF4-dependent manner (213). The study further demonstrated that ATF4-deficient DA neurons were resistant to cell death induced by PD neurotoxins. Importantly, pharmacological inhibition of ATF4 with the eIF2α inhibitor C16 protected against PD-neurotoxin-induced cell death. In contrast, a previous study demonstrated that ATF4 overexpression in the brain by recombinant adeno-associated virus (rAAV) aggravated DA neuron loss in the SNpc in a rat model of PD (296). Deletion of CHOP was shown to exert a neuroprotective effect in a mouse model of PD, particularly during the acute period of PD (47).

Additionally, a previous study demonstrated that XBP1 overexpression exerted protective effects against DA neuronal death induced by PD inducers MPP**^+^** and 1-methyl-4-phenyl-1,2,3,6-tetrahydropyridine (MPTP) (218). Consistently, viral transfection for XBP1 expression in the substantia nigra inhibited DA neuronal degeneration caused by PD-inducing neurotoxins (214). Furthermore, another study in a mouse model of PD demonstrated that XBP1-deficient DA neurons were resistant to PD neurotoxin 6-OHDA, whereas XBP1 overexpression in the SNpc of mice protected DA neurons against 6-OHDA-induced cell death (215). However, in mouse PD models, genetic ablation of ATF6 has been shown to present with accelerated DA neuronal death induced by PD neurotoxins (216, 217).

Collectively, these findings highlight the importance of ER stress in the physiopathology of PD and indicate that ER stress plays a vital role in inhibiting DA neuronal loss and degeneration induced by various PD neurotoxins. Pharmacological or genetic targeting of ER stress markers may represent a potential mechanism for the development of therapeutic strategies against PD.

### The Role of Endoplasmic Reticulum Stress in Multiple Sclerosis

Multiple sclerosis (MS) is a chronic autoimmune-mediated inflammatory disease of the CNS characterized by demyelination with concomitant axonal and neuronal degeneration (297). While the etiology of MS remains unknown, it is believed to be initiated by autoreactive T lymphocytes that have crossed the BBB and triggered an autoimmune response by self-CNS antigens (298). ER stress induced by the accumulation of misfolded proteins is a hallmark of MS pathology (299). Microarray analysis showed that the levels of the ER stress markers ATF4 and heat shock protein 70 in MS demyelinated lesions were highly upregulated (300, 301). Furthermore, the levels of the ER stress markers GRP78, ATF4, and CHOP were found to be significantly upregulated in the white matter of MS patients compared with that in non-MS individuals (302). Consistent with this observation, detailed semiquantitative immunohistochemical and molecular analyses of multiple CNS cell types in biopsy specimens and in postmortem samples revealed that GRP78, XBP1, and CHOP were significantly increased in astrocytes, microglia, and oligodendrocytes in MS lesions (303–305). Studies in an experimental autoimmune encephalomyelitis (EAE) model of MS indicated that ER stress regulates oligodendrocyte viability during EAE and subsequently influences MS progression (219, 223, 306). A prominent MAM regulatory protein, guanosine triphosphatase (GTPase) Rab32, was reported to be correlated with ER stress proteins in the MS brain; it connects ER stress to mitochondrial dysfunction in MS (307). Alternatively, upregulation of human endogenous retrovirus (HERV) envelop proteins may aggravate MS pathology by triggering ER stress and neuroinflammation in the brain (308, 309).

Interferon-γ (IFN-γ) plays a dual role in the pathology of MS (58). CNS-expression of IFN-γ before EAE onset significantly promoted activation of the PERK/eIF2α pathway in oligodendrocytes, preventing oligodendrocyte death, demyelination, and axonal degeneration in the CNS of EAE mice (306). Furthermore, CNS delivery of IFN-γ before EAE onset does not ameliorate the severity of disease course or inhibit EAE-induced oligodendrocyte death, demyelination, and axonal degeneration in PERK-deficient mice. These results demonstrate that the beneficial role of IFN-γ in EAE is dependent on PERK activation. However, several studies have demonstrated that CNS expression of IFN-γ during development promotes myelinating oligodendrocyte death, hypomyelination, and inflammation (310–312). Interestingly, CNS expression of IFN-γ activates the PERK/eIF2α pathway in myelinating oligodendrocytes, and the increased expression of IFN-γ in PERK-deficient mice dramatically promoted CNS hypomyelination and enhanced oligodendrocyte death (313, 314). These results indicate that PERK plays a neuroprotective role in MS progression, regardless of whether IFN-γ expression is beneficial or deleterious in immune-mediated demyelination. Subsequent studies further validated these results and concluded that moderate PERK activation exerts protective effects on oligodendrocytes in a model of MS. In a mouse model (PLP/Fv2E-PERK mice) that allows for temporally controlled activation of PERK signaling specifically in oligodendrocytes, moderate PERK activation in oligodendrocytes significantly attenuated the EAE disease course, which was associated with reduced oligodendrocyte loss, demyelination, and axonal degeneration (219). Similarly, in the same mouse model for MS and EAE, another study showed that enhancing PERK activation in oligodendrocytes protects the cells and myelin against the deleterious effects of IFN-γ (220). Consistent with this result, oligodendrocyte (OL)-specific PERK-knockout (OL-PERK ^ko/ko^) mice were reported to be susceptible to EAE (223). These mice exhibited a significantly more severe EAE disease course, which was associated with oligodendrocyte loss, demyelination, and axonal degeneration (223). A recent study confirmed that PERK activation specifically in oligodendrocytes significantly prevented neuronal loss in the CNS of EAE mice (221). Further investigation demonstrated that ATF4 inactivation specifically in oligodendrocytes did not alter EAE disease severity and did not significantly affect oligodendrocyte loss, demyelination, or axonal degeneration in the CNS of EAE mice. Consistent with this finding, a previous report demonstrated that CHOP deficiency did not influence the development of EAE (308). Although moderate PERK activation in oligodendrocytes is generally believed to protect oligodendrocytes (both mature and remyelinating oligodendrocytes) against inflammatory attacks in immune-mediated demyelinating diseases, a recent study has revealed that PERK may exert deleterious effects on the development of MS. This study showed that ER stress is involved in inflammation and astrogliosis primarily *via* the PERK/JAK1/STAT3 pathway in the MS mouse model of EAE (112). In addition, modulation of ER stress by inhibition of eIF2α/CHOP and activation of XBP1 prevented optic neuritis in MS (224). ATF6, another branch of the UPR, has recently been implicated in the development of MS. A study revealed that ATF6α deficiency increased the sensitivity of myelinating oligodendrocytes to IFN-γ-induced ER stress and exacerbated EAE disease severity, which was associated with increased oligodendrocyte death and myelin loss (222).

Collectively, these findings highlight the importance of ER stress in the pathology of MS and indicate that ER stress may exert a neuroprotective role in EAE onset and development. Of the three key sensors of the UPR, the role of PERK signaling in oligodendrocytes has been the best described. Although the IRE1α/XBP1 pathway is the most conserved branch of the UPR, there is no evidence indicating that this pathway plays a vital role in oligodendrocytes or even in the pathology of MS.

### The Role of Endoplasmic Reticulum Stress in Huntington Disease

Huntington disease (HD) is a rare but fatal autosomal dominant neurodegenerative disease caused by a repeat CAG expansion, encoding polyglutamine (polyQ) stretch in the huntingtin (Htt) gene, resulting in motor and cognitive deficits that are progressively disabling (315). In HD, expansion of polyQ stretch within the first exon of Htt results in mutant Htt (mHtt) misfolding aggregation. Emerging evidence has identified that accumulation of mHtt in the ER and the concomitant induction of ER stress play a crucial role in the pathology of HD (226, 316–319). Postmortem evidence has shown that ER stress markers, such as BiP and CHOP, are upregulated in HD human brains (320). Several *in vivo* and *in vitro* experimental studies have revealed that modulation of ER stress may be an attractive approach to reduce cellular toxicity and identify a potential therapeutic target for the treatment of HD.

A previous study showed that GRP78 overexpression significantly protected N2a cells against mHtt, reduced the formation of mutant huntingtin aggregates, and prevented caspase-12-mediated cell death (225). An *in vitro* study aimed at revealing the role of eIF2α phosphorylation status in striatal cell death showed that the PERK inhibitor A4 considerably reduced, in a dose-dependent manner, the additional cytotoxicity in polyQ-expanded Htt (STHdh ^Q111/111^) cells to the level in the full-length wild-type (WT) Htt form (STHdh ^Q7/7^) cells (226). In addition, salubrinal, an inhibitor of ER stress, has been reported to reduce the accumulation of mHtt by upregulation of BiP and p-eIF2α, preventing caspase-12-mediated PC6.3 cell death (227). Additionally, IRE1α has been reported to play a vital role in the ER stress-mediated aggregation of mutant huntingtin. Recently, ubiquitin-specific protease-14 (Usp14), which can directly interact with IRE1α, was reported to reduce cellular aggregates in mHtt-expressing cells and protect against cell degeneration and caspase-3-mediated cell death primarily by inhibiting the phosphorylation of IRE1α (228). IRE1α knockdown by shRNA significantly reduced ER stress-induced mHtt toxicity (229). In a study that used AAV-mediated delivery of XBP1 into the stratum of mice, the accumulation of mHtt inclusion was dramatically reduced when XBP1 was co-expressed in the stratum (230). In addition, XBP1 deficiency in the nervous system significantly decreased the levels of mHtt in the striatum of HD transgenic mice (231). In this study, shRNA-mediated knockdown of both IRE1α and XBP1 in two neuronal cell lines showed a significant reduction in the aggregation of pathological polyQ79-EGFP peptides. Furthermore, ATF5, as a part of the UPR, is decreased and sequestered into polyQ inclusions in HD. Decreased ATF5 levels exacerbated polyQ toxicity *in vivo*. Conversely, overexpression of ATF5 inhibited polyQ-induced apoptosis in a cell model of HD (321).

Collectively, these studies indicate that ER stress plays an important role in regulating the cellular aggregation of mHtt and protecting cells against cellular toxicity during HD. Targeting ER stress responses may be a mechanism to ameliorate motor and cognitive deficits in HD.

### The Roles of Endoplasmic Reticulum Stress in Amyotrophic Lateral Sclerosis

Amyotrophic lateral sclerosis (ALS) is a devastating adult-onset neurodegenerative disease characterized by progressive degeneration of both upper and lower motoneurons, which results in muscle weakness, paralysis, and eventual death (322). Currently, no effective drugs have been demonstrated to be effective against ALS. Emerging evidence supports the concept that the accumulation of neurotoxic misfolded proteins, inclusions, and aggregates in motoneurons is the main pathological hallmark of ALS (323). These protein aggregates are believed to disrupt cellular proteostasis and eventually lead to ER stress within the motoneuron. Postmortem evidence has shown that the expression of the UPR pathway is significantly upregulated in the spinal cords of patients with sporadic or familial ALS (324–328). In addition, structural changes associated with ER stress, such as fragmentation, were detected in the anterior horn of the spinal cord in ALS, and several ER stress markers were observed in the cerebrospinal fluid (CSF) of patients with sporadic ALS patients (328–330). Notably, mutant Cu, Zn-superoxide dismutase (mtSOD1) has been reported to directly interact with GRP78, which upregulates GRP78 expression in ALS mice before the onset of motor symptoms (331). Furthermore, a recent study indicated that ER stress is responsible for the accumulation of wild-type SOD1 aggregates in sporadic ALS (332).

Emerging evidence has confirmed that ER stress is implicated in the onset and progression of ALS and that targeting the UPR/integrated stress response (ISR) may serve as a strategy for ameliorating motor symptoms and extend the lifespan in ALS (333).

The ER stress inhibitor salubrinal was reported to ameliorate disease severity and delay progression in ALS (232). Furthermore, *in vitro* experiments demonstrated that salubrinal protected Neuro2a cells against mtSOD1-induced cell death (233). In a G85R mtSOD1 transgenic mouse model of ALS, compared to PERK^+/+^ mice, PERK^+/-^ mice exhibited an earlier disease onset, reduced lifespan, and earlier neuropathological alterations in the spinal cord (234). In parallel with this finding, in the same ALS model, mice with GADD34 dysfunction (GADD34^ΔC^) exhibited delayed disease onset, delayed early phase of disease, and prolonged lifespan compared with the control littermate G85R mice (235). Consistent with this result, GADD34 knockdown, using AAV-mediated delivery of GADD34 shRNA into G85R mtSOD1 mice, was found to significantly ameliorate disease severity and prolong lifespan in mice (236). Guanabenz, an inhibitor of GADD34-mediated dephosphorylation of p-eIF2α, has been reported to ameliorate disease severity with a delay in the onset and prolongation of the early phase of disease and survival of mtSOD1 transgenic mice (237). Further validation showed that guanabenz ameliorated disease severity by attenuating proapoptotic protein-mediated neuron loss, which was associated with delayed onset of disease symptoms, prolonged lifespan, and improved motor performance (238). TAR DNA binding protein 43 (TDP-43) links both familial and sporadic forms of ALS as mutations are the leading cause of disease, and cytoplasmic aggregates are a pathological hallmark of almost all cases (334). Importantly, recent studies indicate that mutant TDP-43 may be driven by the activation of ER stress in motor neurons (335–337). Recently, both guanabenz and salubrinal have been reported to ameliorate motor deficits and axon defects, which are associated with mutant TDP-43-induced toxicity in the mutant TDP-43 model of ALS (240). However, in contrast to these observations, a later study demonstrated that guanabenz treatment could accelerate the progression of ALS-like disease in mtSOD1 transgenic mice (338). In general, these observations suggest that GADD34, a protein downstream of the PERK/eIF2α pathway, plays a vital role in the onset and progression of ALS. Additionally, both *in vivo* and *in vitro* experiments demonstrated that the PERK inhibitor GSK2606414 effectively inhibited ER-stress-associated TDP-43 toxicity (241). Another PERK inhibitor, ISRIB, was reported to effectively decrease G93A SOD1-mediated neuronal death by regulating the ER stress response (239).

Collectively, these findings indicate the potential therapeutic role of ER stress in ALS. Pharmacological or genetic targeting of ER stress has been reported to ameliorate disease severity and improve ALS symptomatology in animal experiments. Nevertheless, of the three key sensors of the UPR, the role of PERK signaling in ALS has been the best described. Therefore, further investigations are needed for demonstrating the role of IRE1α and ATF6 in the alteration of ALS symptomatology.

## Conclusion and Future Perspective

**Figure 3:**
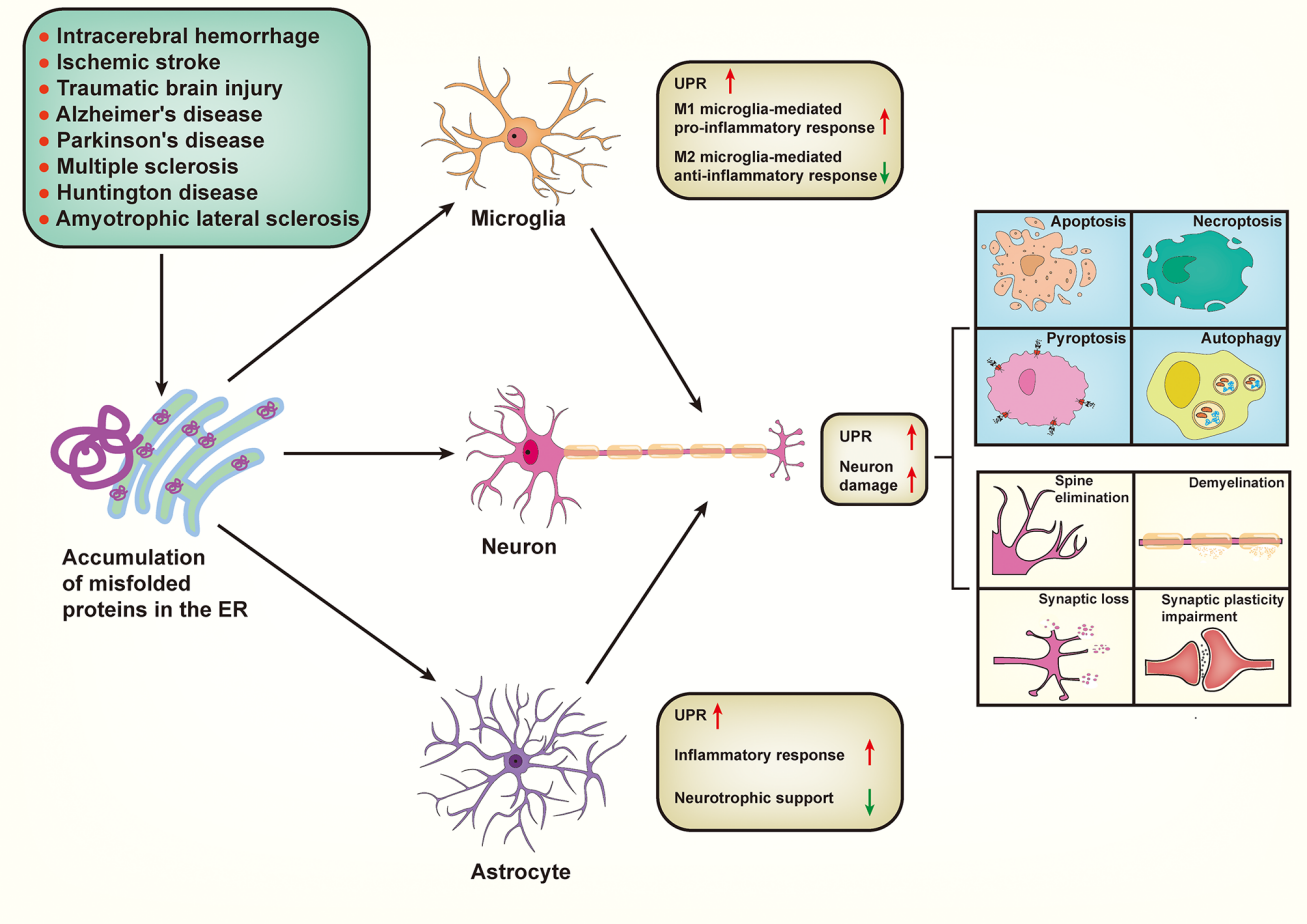
Proposed mechanism by which endoplasmic reticulum stress signaling impacts the overall central nervous system envirment. Various neurological diseases share a common pathogenesis, that is aberrant accumulation of misfolded proteins within the endoplasmic reticulum (ER). Pathologically, accumulation of misfolded proteins in ER subsequently triggers ER stress and concomitant unfolded protein response (UPR) in microglia, astrocytes, and neurons in various neurological diseases. ER stress occurred in neurons can trigger UPR, resulting in a series of neuronal damage including cell death (apoptosis, necroptosis, pyroptosis, and autophagy), spine elimination, demyelination, synaptic loss, and synaptic plasticity impairment. In addition, UPR-activated microglia promote the polarization of microglia from pro-inflammatory M1 phenotype to anti-inflammatory M2 phenotype. Similarly, UPR-activated astrocytes exhibit an increase of inflammatory response but a decrease of neurotrophic support. Importantly, both microglia-mediated and astrocyte-mediated inflammatory responses can “transmit” ER stress to neurons, thereby aggravating neuronal damage.

In this review, we comprehensively discuss the underlying mechanism of ER stress in neuron damage and immune responses in the CNS, emphasizing their pivotal roles in the pathogenesis of various neurological diseases and potential therapeutic strategies (**Figure 3**).

Currently, there is no effective therapeutic approach in clinical treatments that can improve the prognosis of most neurological diseases. Therefore, it is urgent to identify novel therapeutic targets to modulate disease-associated signaling cascades. Pathologically, neuronal cell death and the inflammatory response are the two most common hallmarks of acute CNS injury and chronic degenerative disorders. Importantly, myriad clinical trials and preclinical animal experiments have indicated that the aggregation and accumulation of proteins and the concomitant induction of ER stress are closely associated with neuronal death and neuroinflammation in many neurological diseases. The induction of the UPR by ER stress is a sophisticated and coordinated biochemical response that is aimed to maintain cellular proteostasis, whereas its hyperactivation is closely associated with cell death and immune response. Thus, understanding how to control the balance between the physiological UPR and the pathological UPR in many disease states, and how this delicate balance can be manipulated to restore homeostasis to enable appropriate cell death and immune responses, are essential directions for further investigation.

To date, numerous preclinical studies have demonstrated the potential therapeutic effects of the pharmacological targeting of ER stress in animal models of neurological diseases. However, the pharmacological targeting of ER stress remains challenging, as the response is broadly engaged in the physiopathology of various cell types and organs, such as the liver and heart, so the long-term administration of drugs may lead to serious adverse effects. Genetic targeting of the ER has gradually been placed as an attractive and therapeutic alternative to drugs because it can selectively target specific brain regions. Nevertheless, both the pharmacological and the genetic targeting of ER stress as therapeutic approaches for neurological disease remain limited to preclinical animal studies with no clinical evidence. Therefore, further efforts are needed to translate such work into clinical studies.

## Author Contributions

MS searched the bibliography and drafted the manuscript. YC prepared the figures. JZ and XC critically revised the manuscript. All authors contributed to the article and approved the submitted version.

## Funding

This work was supported by grants from the National Natural Science Foundation of China (no. 816719029), and the Project of Tianjin Applied Basic and Cutting-edge Technological Research (17JCYBJC25200) and by the Tianjin Health Care Elite Prominent Young Doctor Development Program and the Young and Middle-aged Backbone Innovative Talent Program.

## Conflict of Interest

The authors declare that the research was conducted in the absence of any commercial or financial relationships that could be construed as a potential conflict of interest.

## Publisher’s Note

All claims expressed in this article are solely those of the authors and do not necessarily represent those of their affiliated organizations, or those of the publisher, the editors and the reviewers. Any product that may be evaluated in this article, or claim that may be made by its manufacturer, is not guaranteed or endorsed by the publisher.
